# Predicting pathological complete response following neoadjuvant chemoradiotherapy (nCRT) in patients with locally advanced rectal cancer using merged model integrating MRI-based radiomics and deep learning data

**DOI:** 10.1186/s12880-024-01474-3

**Published:** 2024-10-24

**Authors:** Haidi Lu, Yuan Yuan, Minglu Liu, Zhihui Li, Xiaolu Ma, Yuwei Xia, Feng Shi, Yong Lu, Jianping Lu, Fu Shen

**Affiliations:** 1 Department of Radiology, Changhai Hospital, Naval Medical University, 168 Changhai Road, Shanghai, 200433 China; 2 Department of Radiology, RuiJin Hospital LuWan Branch, Shanghai Jiaotong University School of Medicine, Shanghai, China; 3 Shanghai United Imaging Intelligence, Co., Ltd, Shanghai, China; 4 Department of Radiology, RuiJin Hospital, Shanghai Jiaotong University School of Medicine, 197 Ruijin Er Road, Shanghai, 200025 China

**Keywords:** Rectal cancer, Neoadjuvant therapy, Radiomics, Magnetic resonance imaging, Deep learning

## Abstract

**Background:**

To construct and compare merged models integrating clinical factors, MRI-based radiomics features and deep learning (DL) models for predicting pathological complete response (pCR) to neoadjuvant chemoradiotherapy (nCRT) in patients with locally advanced rectal cancer (LARC).

**Methods:**

Totally 197 patients with LARC administered surgical resection after nCRT were assigned to cohort 1 (training and test sets); meanwhile, 52 cases were assigned to cohort 2 as a validation set. Radscore and DL models were established for predicting pCR applying pre- and post-nCRT MRI data, respectively. Different merged models integrating clinical factors, Radscore and DL model were constituted. Their predictive performances were validated and compared by receiver operating characteristic (ROC) and decision curve analyses (DCA).

**Results:**

Merged models were established integrating selected clinical factors, Radscore and DL model for pCR prediction. The areas under the ROC curves (AUCs) of the pre-nCRT merged model were 0.834 (95% CI: 0.737–0.931) and 0.742 (95% CI: 0.650–0.834) in test and validation sets, respectively. The AUCs of the post-nCRT merged model were 0.746 (95% CI: 0.636–0.856) and 0.737 (95% CI: 0.646–0.828) in test and validation sets, respectively. DCA showed that the pretreatment algorithm could yield enhanced clinically benefit than the post-nCRT approach.

**Conclusions:**

The pre-nCRT merged model including clinical factors, Radscore and DL model constitutes an effective non-invasive tool for pCR prediction in LARC.

**Supplementary Information:**

The online version contains supplementary material available at 10.1186/s12880-024-01474-3.

## Background

Patients with locally advanced rectal cancer (LARC) are routinely administered neoadjuvant chemoradiotherapy (nCRT) combined with total mesorectal excision (TME) [1, 2]. Approximately 15–27% cases achieve pathological complete response (pCR) and are expected to undergo “Watch & Wait” or nonoperative management (NOM) [3]. The goal of an organ-preserving approach is to maintain quality of life while ensuring safety, without early recurrence or impact on overall survival. In comparison with surgery, total survival time shows no significant difference, while effectively reducing surgical complications and mortality [4, 5]. However, variable response to nCRT has been reported, with some LARC cases showing no obvious tumor regression but suffering from side effects, which hinders the administration of timely surgery or other systemic treatments. Therefore, accurate prediction of pCR may enhance treatment personalization and efficacy. However, the final confirmation of pCR status can solely be made by post-surgical histopathologic examination [6]. Accordingly, developing a noninvasive pretreatment tool to determine candidates for an organ-preserving approach is highly critical.

Nowadays, rectal high-resolution magnetic resonance imaging (mainly including T2WI) is routinely performed for clinical imaging to identify individuals showing a good response and suitability for NOM [6–8]. The Magnetic Resonance Imaging and Rectal Cancer European Equivalence (MERCURY) study group developed an MRI-based Tumor Regression Grading (mrTRG) tool according to the five-category pathological tumor regression grading (pTRG) scale [6]. However, currently reported findings demonstrated this approach is defective because of distinct categories and variable subjective judgements. Therefore, a robust noninvasive tool for accurate identification of patients with pCR before or after nCRT needs to be developed. MRI-based radiomics has many advantages in the assessment of treatment response versus traditional imaging evaluation [7], representing a high-value noninvasive tool for predicting pCR status in LARC [8–12]. Meanwhile, substantial evidence reveals a potential benefit for MRI-based deep learning (DL) models for assessing therapeutic response to nCRT, with higher significance than subjective evaluation [13–16]. Among them, previous findings demonstrated the clinical benefits of clinical factors, MRI radiomics or DL models based on pre- and/or post-nCRT T2WI sequences in assessing therapeutic response in LARC [17, 18].

To our knowledge, however, the radiomics and DL models based on pre- or post-nCRT MRI that can yield enhanced clinically benefits remain unknown. We hypothesized that a combined model applying MRI radiomics features, DL model and clinical parameters might constitute a more reliable tool. Therefore, the current work aimed to validate and compare pre-nCRT and post-nCRT merged models in the prediction of pCR following nCRT for LARC.

## Methods

### Patients

This study followed the Declaration of Helsinki and had approval from the ethics committee of Changhai Hospital (B2023-022), who waived the requirement for informed consent due to a retrospective design.

First, patients with clinical diagnosis of LARC and administered TME upon nCRT in Changhai Hospital from January 2018 to December 2023 were retrospectively analyzed for model building (cohort 1). Next, LARC cases with the same inclusion criteria treated from June 2019 to December 2023 in Ruijin Hospital Luwan branch were examined for validation (cohort 2).

Inclusion criteria were: (1) initial endoscopic biopsy revealing histologically confirmed rectal adenocarcinoma; (2) primary rectal cancer confirmed as stage II (T3-4N0M0) or stage III (T1-4N1-2 M0) by clinical assessment; (3) pre-nCRT MRI within 14 days before nCRT; (4) post-nCRT MRI were routinely performed within 7 presurgical days; (5) single focus tumor.

Exclusion criteria were: (1) previously diagnosed malignancy or pelvic surgery (*n* = 7); (2) chronic inflammatory bowel disease (*n* = 5); (3) poor image quality or no high-resolution T2WI (*n* = 11); (4) nCRT and rectal surgery separated by more than 12 weeks (*n* = 19); (5) preoperative concomitant intestinal obstruction or perforation (*n* = 7). Altogether, the current study eventually included 197 and 52 cases in cohorts 1 and 2, respectively. Cohort 1 cases with the corresponding pCR statuses were randomized into the training and test sets at 8:2.

Baseline clinical features, including age, gender, BMI, pre-nCRT serum carcinoembryonic antigen (CEA, ng/mL, within 1 month of MRI), interval between nCRT to surgery, and interval between post-nCRT MRI to surgery were retrieved from medical records.

### Image acquisition and subjective MRI evaluation

Rectal MR images were obtained pre- and post-nCRT, respectively, on a 3.0T or 1.5T MR scanner with an abdominal phase array coil. The patients underwent a 4-h fasting prior to MRI. Before MRI, enema was administered with glycerin (20 ml) to clean the intestines. Rectal high-resolution axial oblique T2W sequences were applied to obtain images perpendicular to the tumor’s long axis and/or post-nCRT lesion area, and data were used for both radiomics analysis and DL modeling. Supplementary Table S1 summarizes the parameters used for high-resolution T2W sequence.

Pre-nCRT MRI factors [19], including tumor height to anal margin, MR-based T and N stages, mesorectal fascia (MRF) and extramural vascular invasion (EMVI), were assessed by two radiologists (Y.Y. and HD.L. with 12 and 10 years of experience in MRI evaluation, respectively) on a GE PACS RA1000 image archiving and communication system workstation. Each radiologist was blinded to histopathologic data and treatment outcomes, except for the diagnosis of rectal cancer. Any discrepancy between the above two radiologists was resolved by consensual discussion.

### Neoadjuvant treatment

The cases were administered neoadjuvant treatment with long-course pelvic radiotherapy (50.4 Gy in 25 to 28 fractions) and oral capecitabine (825 mg/m^2^ b.i.d.). After 8–12 weeks, radical surgery was carried out.

### Definition of tumor regression

Referring to the National Comprehensive Cancer Network and American Joint Committee on cancer staging system [20], tumor regression grades were recorded as proposed previously [21], and response description utilized the pTRG system. Pathological response grading employed a scale ranging from 0 for complete response (no viable cancer cells) to 3 for poor response (negligible or no cancer cell killing). Pathological complete response (pCR) was reflected by the absence of viable cancer cells in primary tumors or lymph nodes (ypT0N0M0).

### Clinical factor analysis

Univariable logistic regression analysis was carried out in the training set for various clinical factors such as gender, age, BMI, tumor height, MR T and N stages, MRF, EMVI, and CEA to select indexes independently predicting pCR.

### Radiomics analysis

High-resolution T2W DICOM images originally obtained before and after nCRT, respectively, were imported into the uAI Research Portal (United Imaging Intelligence, Shanghai, China).

**Fig. 1:**
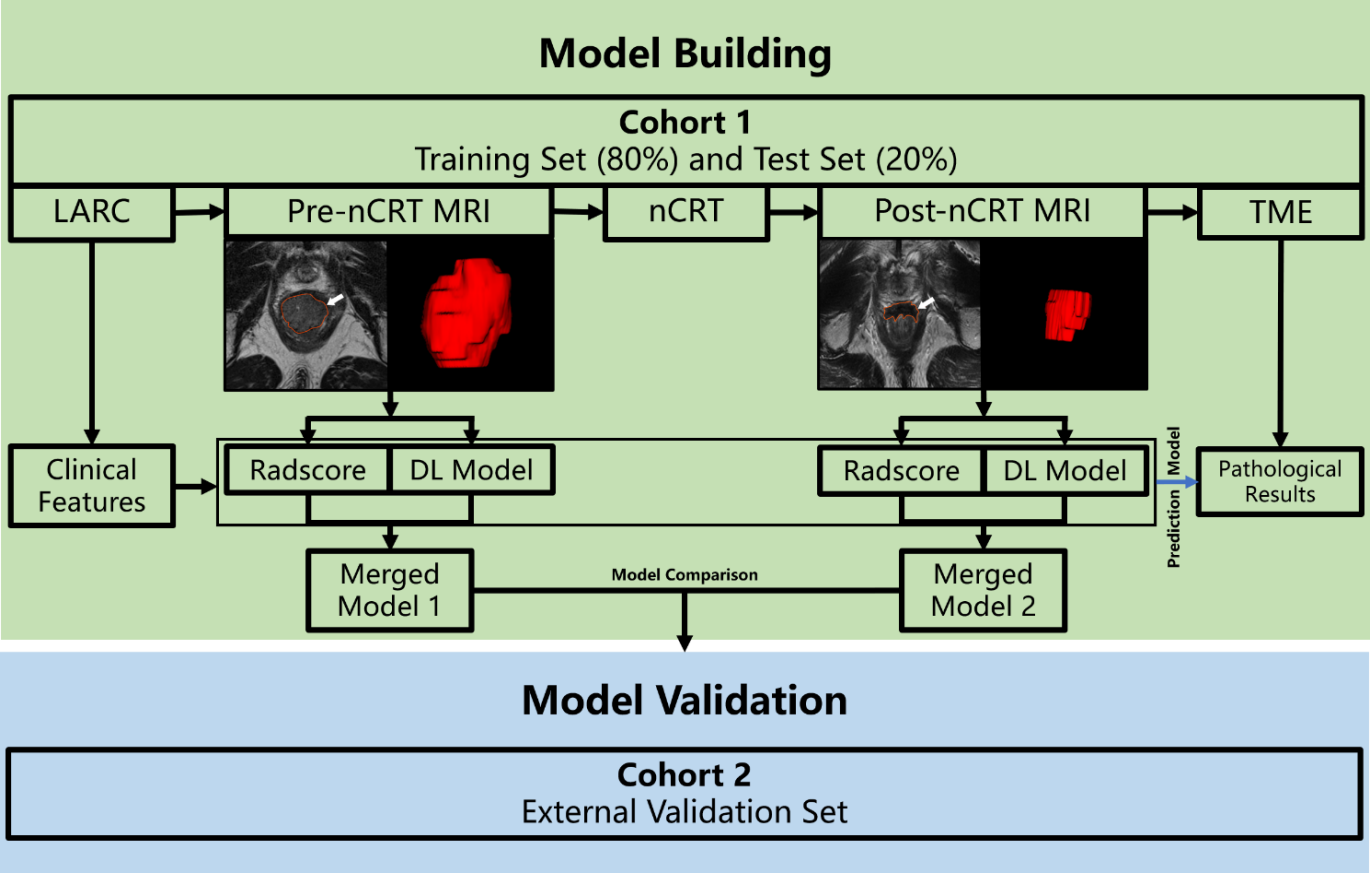
Study flowchart, including model building, comparison, representative images for lesion contouring and the validation process. Center 1: Changhai Hospital; Center 2, Ruijin Hospital Luwan Branch. nCRT: neoadjuvant chemoradiotherapy; LARC: locally advanced rectal cancer; TME: Total mesorectal excision; DL: deep learning

One radiologist (HD.L., 10 years of experience in MRI evaluation) performed a manual segmentation of the region of interest (ROI) on each pre- and post-CRT T2WI scan (Fig. 1). For segmentation in baseline MRI, a ROI was delineated along the tumor margin not including the outer non-rectal tissue and normal rectum. For segmentation in post-CRT MRI, baseline MRI was employed to identify the initial tumor location, and a ROI was drawn covering the entire post-nCRT lesion including signal intensity demonstrating fibrosis or mucin.

Subsequently, 2264 radiomics features were automatically retrieved from the uAI Research Portal. The various features defined below comply with the Imaging Biomarker Standardization Initiative (IBSI), subdivided into the following classes: (1) first-order statistics; (2) 2D and 3D shape-based features; (3) texture properties (gray-level cooccurrence matrix, gray-level run length matrix, gray-level size zone and neighborhood gray-tone difference matrices, and gray-level dependence matrix features); (4) higher-order statistics (first-order statistics and texture features post-transformation) [22–24]. Before training the model, data preprocessing was performed to normalize the original radiomics data by Z–score scaling. In addition, both radiologists (Y.Y. and HD.L.) performed segmentations a second time for 30 cases randomly chosen from the pre- and post-nCRT datasets a month later. Inter- and intraclass correlation coefficients (ICCs) were determined to evaluate inter-observer reliability and intra-observer reproducibility for each feature. Features with inter- and intra-observer ICCs > 0.8 (good robustness) were further examined. Next, the variance threshold algorithm (variance threshold = 0.1) was applied for further reduction. At last, the least absolute shrinkage and selection operator (LASSO) algorithm was utilized to determine optimal parameters related to pCR. Leave-one-out cross-validation was employed for selecting the optimal regularization index alpha, as average mean square errors for various patients were small. Features with non-zero coefficients were employed to build Radscore-1 based on the training set of pre-nCRT MRI data and Radscore-2 trained using the post-nCRT dataset.

### DL model construction

To facilitate pCR prediction, the simple and easily manageable ResdualNet was introduced to classify 3D ROIs [25–27]. The residual block was considered the basic unit for the construction of a neural network for classification in the training set. The details of DL model are provided in Supplementary Table S2. Segmentation data from radiomics analysis were trained as the input for classification. Two DL models were constructed, with DL model-1 trained on pre-nCRT MRI data and DL model-2 trained exclusively on the post-nCRT dataset.

### Merged models

A multivariate logistic regression analysis combining the selected clinical features, Radscore and DL-model predicted probability was carried out to establish a prediction model for pCR. Merged model 1 encompassed clinical factors, Radscore-1 and DL model-1 in the pre-nCRT dataset. Merged model 2 included clinical factors, Radscore-2 and DL model-2 in the post-nCRT dataset. Figure 1 shows the study flowchart.

### Statistical analysis

Continuous variates, expressed as mean ± standard deviation, were assessed for normality by the Kolmogorov-Smirnov test. Those with normal and skewed distributions were compared by the Student’s t-test and the Kruskal-Wallis H test, respectively. Categorical variates were compared by the Pearson Chi-square test or the Fisher’s exact test. Receiver operator characteristic (ROC) curves were generated to examine the performances of the obtained models by determining areas under the ROC curves (AUCs) in both datasets. The models were compared by the DeLong’s test. The diagnostic sensitivity, specificity, accuracy, precision and F1 score were assessed for each model. The confusion matrix and decision curve analysis (DCA) were performed to assess the models’ clinical potentials. Calibration curve analysis and the Brier score test were utilized for goodness of fit estimation. Two-sided *P* < 0.05 suggested statistical significance. MedCalc 19.8 (MedCalc Software, Mariakerke, Belgium) and R 4.1.3 (Vienna, Austria) were employed for data analysis.

## Results

### Patient characteristics

Totally 197 and 52 patients were assessed in cohorts 1 and 2, respectively. Both cohorts were comparable in clinicopathological indexes that are summarized in Table 1. According to postoperative pathological examination, 43 patients (21.8%) showed pCR in cohort 1, and 12 cases (23.1%) were classified as pCR in cohort 2. Univariable logistic regression analysis in the training set revealed CEA, MR T stage and N stage as independent predictive factors of pCR in LARC cases (Supplementary Table S3).

**Table 1:** Patient demographics

Clinicopathological parameter		Cohort 1	Cohort 2	*P* value
		(*n* = 197)	(*n* = 52)	
Gender	%			0.151
	Male	131 (66.5)	29 (55.8)	
	Female	66 (33.5)	23 (44.2)	
Age (years)	Median (IQR)	58.0 (50.0–66.0)	57.0 (51.0-63.5)	0.477
BMI (kg/m^2^)	Median (IQR)	23.7 (21.1–25.5)	24.2 (21.8–26.5)	0.102
Tumor height (cm)	Median (IQR)	5.0 (3.0–6.0)	4.0 (3.0–6.0)	0.765
MR T stage	%			0.097
	T1-2	30 (15.2)	13 (25.0)	
	T3-4	167 (84.8)	39 (75.0)	
MR N stage	%			0.287
	N0	43 (21.8)	15 (28.8)	
	N1-2	154 (78.2)	37 (71.2)	
Pathological T stage	%			0.141
	T0	43 (21.8)	12 (23.1)	
	T1	18 (9.2)	1 (1.9)	
	T2	78 (39.6)	17 (32.7)	
	T3	58 (29.4)	22 (42.3)	
Pathological N stage	%			0.852
	N0	139 (70.6)	36 (69.2)	
	N1-2	58 (29.4)	16 (30.8)	
Pathological TRG	%			0.743
	0	43 (21.8)	12 (23.1)	
	1	35 (17.8)	6 (11.5)	
	2	80 (40.6)	22 (42.3)	
	3	39 (19.8)	12 (23.1)	
pCR	%			0.847
	yes	43 (21.8)	12 (23.1)	
	no	154 (78.2)	40 (76.9)	
CEA	%			0.935
	Negative	130 (66.0)	34 (65.4)	
	Positive	67 (34.0)	18 (34.6)	
MRF	%			0.319
	Negative	132 (67.0)	31 (59.6)	
	Positive	65 (33.0)	21 (40.4)	
EMVI	%			0.160
	Negative	127 (64.5)	28 (53.8)	
	Positive	70 (35.5)	24 (46.2)	
Interval nCRT to surgery, days	Median (IQR)	66.0 (60.0–70.0)	65.5 (58.5–71.5)	0.882
Interval post-nCRT MRI to surgery, days	Median (IQR)	6.0 (4.0–6.0)	5.0 (4.0-6.5)	0.982

nCRT: neoadjuvant chemoradiotherapy; BMI: body mass index; MRF: mesorectal fascia; EMVI: extramural vascular invasion; pCR: pathological complete response; CEA: carcinoembryonic antigen (pre-nCRT blood samples); TRG: tumor regression grade

^*^Tumor height was the distance from the tumor’s lower edge and the anal verge by MRI

Center 1: Changhai Hospital; Center 2, Ruijin Hospital Luwan Branch

### Radiomics and DL models for pCR prediction

**Fig. 2:**
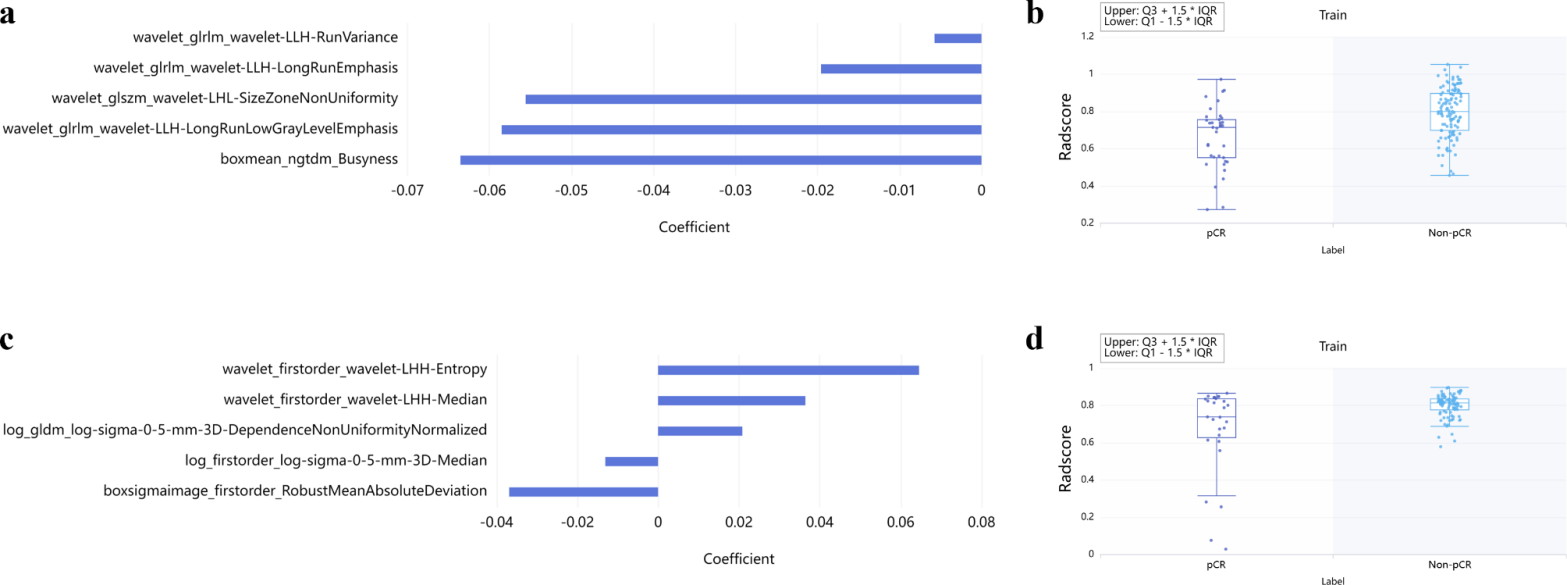
Selected radiomics features and Radscore differences. (**a**) Totally 5 features from Pre-nCRT MRI for pCR status were obtained. (**b**) The difference in Radscore-1 between the pCR and non-pCR groups was significant (*P* < 0.0001). (**c**) Totally 5 features from Post-nCRT MRI for pCR status were obtained. (**d**) The difference in Radscore-2 between the pCR and non-pCR groups was significant (*P* = 0.021)

Totally 2264 radiomics features were derived from rectal MRI data acquired pre- and post-nCRT, respectively. Then, 2037 (90.0%) and 2009 (88.7%) features had good robustness (inter- and intra-observer ICCs above 0.8), respectively, and were further examined. Finally, 5 and 5 vital parameters associated with pCR were selected using the LASSO algorithm (Supplementary Fig. 1) based on rectal MRI data acquired pre- and post- nCRT, respectively, to build Radscore-1 and Radscore-2 (Fig. 2and Supplementary Fig. 2). Significant differences were found between the pCR and non-pCR groups in Radscore-1 (*P* < 0.0001) and Radscore-2 (*P* = 0.021).

ROC analysis of Radscore and DL model in the training set are shown in Supplementary Table S4. In the test set, pre-nCRT Radscore-1 and post-nCRT Radscore-2 had AUCs of 0.713 and 0.653, respectively. DL model-1 from baseline MRI had an AUC of 0.753, and DL model-2 driven from post-nCRT MRI showed an AUC of 0.663. Meanwhile, in the validation set, Radscore-1 and Radscore-2 had AUCs of 0.643 and 0.635, respectively. The AUCs of DL models 1 and 2 were 0.669 and 0.642, respectively. The DeLong’s test showed these models had no significant difference (*P* > 0.05). The models are described in detail in Table 2 and Supplementary Fig. 3. The confusion matrixes are shown in Supplementary Fig. 4.

**Table 2:** ROC curve analysis in the test and validation sets for Radscore and DL model

	Test set				Validation set			
	Radscore-1	DL model-1	Radscore-2	DL model-2	Radscore-1	DL model-1	Radscore-2	DL model-2
AUC	0.713	0.753	0.653	0.663	0.643	0.669	0.635	0.642
95% CI	0.555–0.872	0.602–0.905	0.451–0.856	0.483–0.844	0.452–0.834	0.477–0.861	0.439–0.832	0.458–0.825
Sensitivity	0.833	0.833	0.967	0.833	0.925	0.925	0.875	0.875
Specificity	0.300	0.400	0.100	0.300	0.250	0.167	0.167	0.250
Accuracy	0.700	0.725	0.750	0.700	0.769	0.750	0.712	0.731
Precision	0.781	0.806	0.763	0.781	0.804	0.787	0.778	0.795
F1 Score	0.806	0.820	0.853	0.806	0.860	0.851	0.824	0.833

Radscore-1 and DL model-1 were derived from baseline MRI; Radscore-2 and DL model-2 were derived from post-nCRT MRI

AUC: area under the curve; DL: deep learning

Compared by the DeLong test; all *P* values > 0.05

### Merged models for pCR prediction

**Fig. 3:**
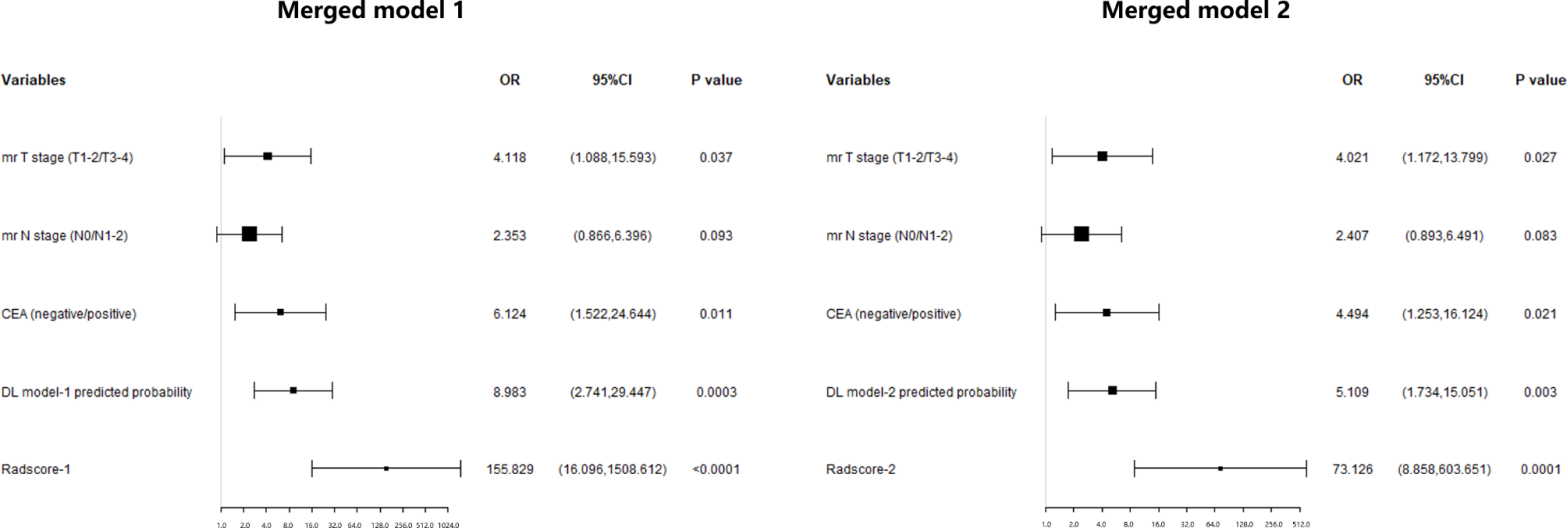
Forest plot depicting multivariate logistic regression analyses for predicting pCR by Radscore, DL model and clinical factors in merged models 1 and 2, respectively. OR: odds ratio; CEA: carcinoembryonic antigen. AUC of Merged model 1: 0.909 (95%CI 0.853–0.949). AUC of Merged model 2: 0.884 (95%CI 0.823–0.929)

In the training set, merged models were established using the multivariate logistic regression analysis (Fig. 3). Merged model 1 including clinical factors, Radscore-1 and DL model-1 showed an AUC of 0.909 (95%CI: 0.853–0.949). Merged model 2 including clinical factors, Radscore-2 and DL model-2 showed an AUC of 0.884 (95%CI: 0.823–0.929). Both models were comparable in the DeLong’s test (*P* = 0.219).

**Fig. 4:**
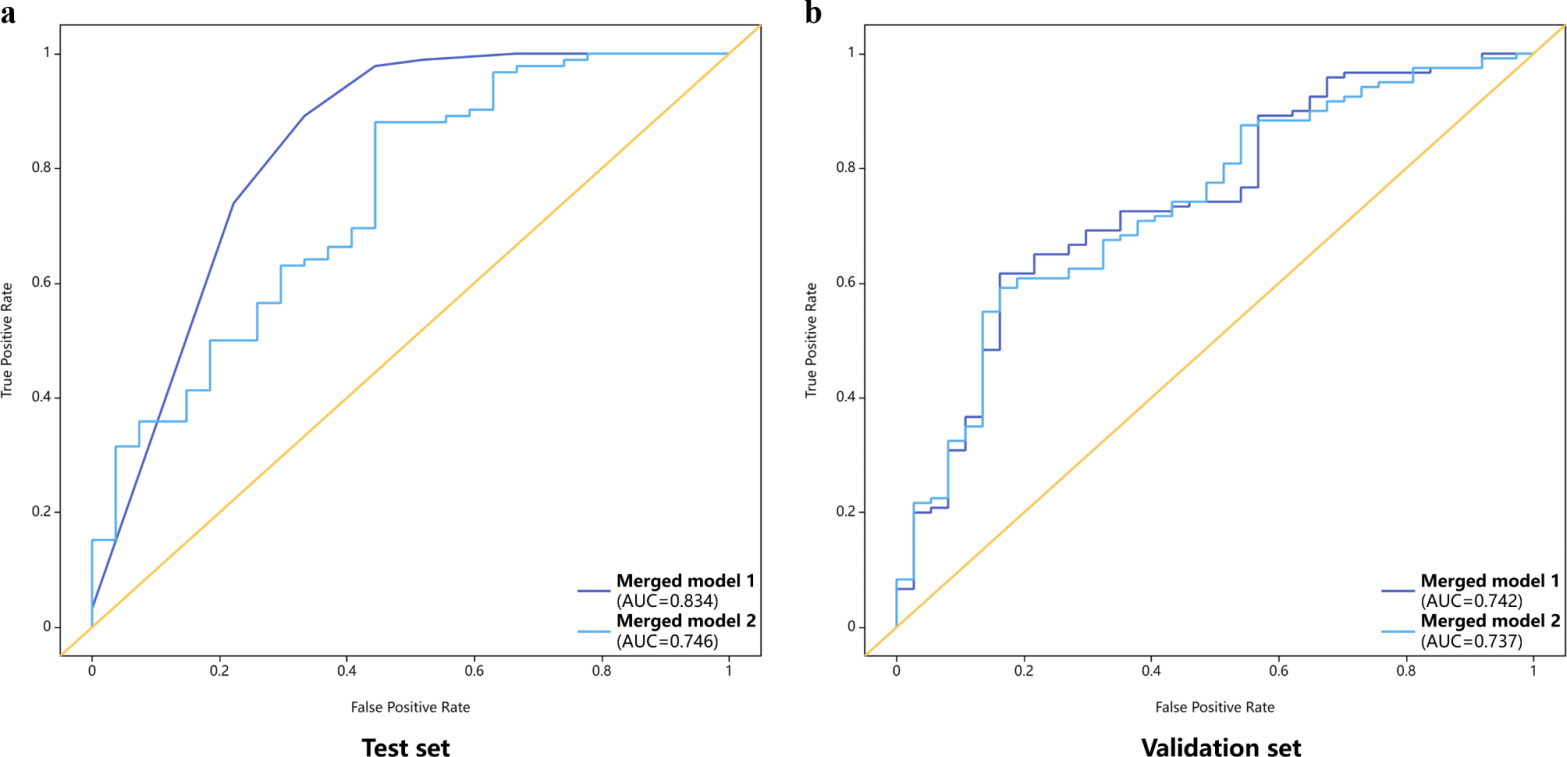
Receiver operator characteristic (ROC) curve analyses of merged models for pCR status prediction. (**a**) In the test set. (**b**) In the validation set

**Fig. 5:**
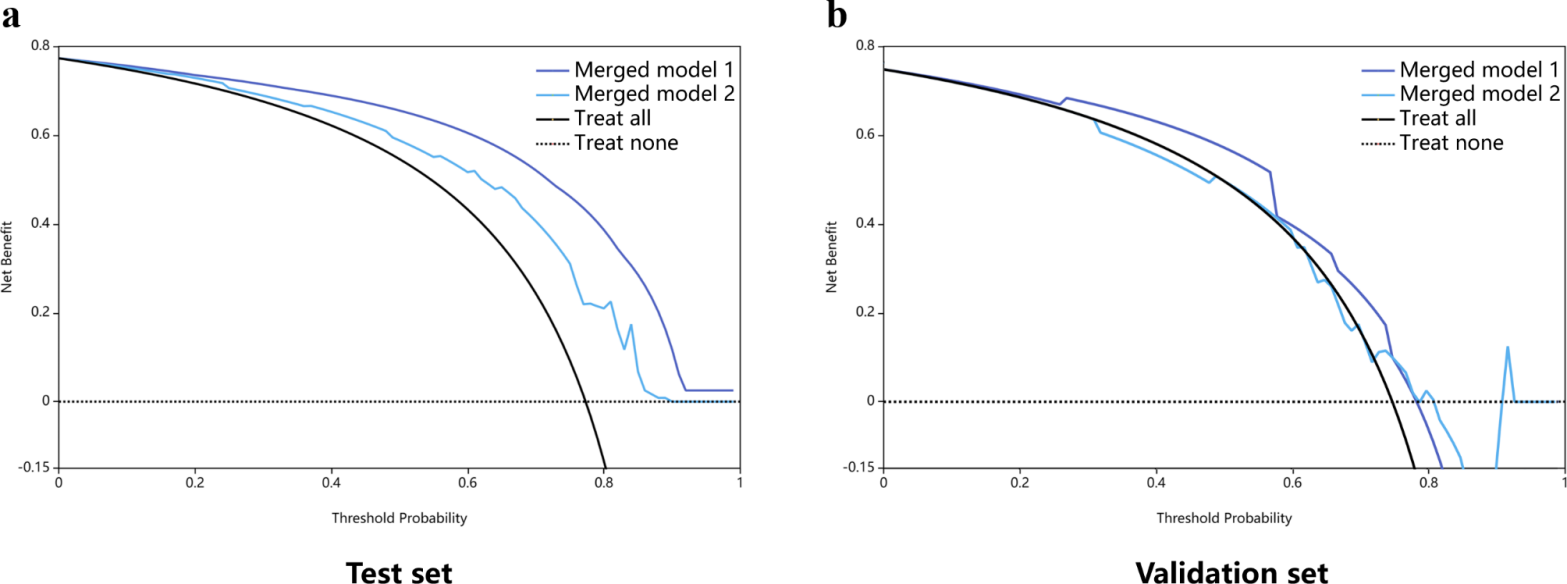
Decision curve analysis (DCA) of the developed merged models. (**a**) In the test set. (**b**) In the validation set

The AUC of merged model 1 was 0.834 (95%CI: 0.737–0.931), with high sensitivity of 97.8%, and an accuracy of 88.2% in the test set; these values were 0.742 (95%CI: 0.650–0.834), 96.7% and 80.9% in the validation set, respectively. Meanwhile, merged model 2 had an AUC of 0.746 (95%CI: 0.636–0.856), with a sensitivity of 97.8% and an accuracy of 81.5% in the test set; these values were 0.737 (95%CI: 0.646–0.828), 85.0% and 75.8% in the validation set, respectively. Compared to merged model 2, the net reclassification improvement index (NRI) of merged model 1 were 0.052 and 0.041 in the test and validation sets, respectively. These models are described in detail in Table 3; Fig. 4. Calibration curves were satisfactory in both sets (Supplementary Fig. 5). DCA demonstrated that merged model 1 had enhanced net benefit compared with merged model 2 at a large threshold probability in both test and validation sets (Fig. 5). In the test and validation sets, DCA showed enhanced net benefit for merged model 1 compared with merged model 2, the treat-all scheme and the treat-none scheme at a wide range of threshold probabilities.

**Table 3:** ROC curve analysis of merged models in the test and validation sets

	Test set		Validation set	
	Merged model 1	Merged model 2	Merged model 1	Merged model 2
AUC	0.834	0.746	0.742	0.737
95% CI	0.737–0.931	0.636–0.856	0.650–0.834	0.646–0.828
Sensitivity	0.978	0.978	0.967	0.850
Specificity	0.556	0.259	0.297	0.459
Accuracy	0.882	0.815	0.809	0.758
Precision	0.882	0.818	0.817	0.836
F1 Score	0.928	0.891	0.885	0.843

Radscore-1 and DL model-1 were derived from baseline MRI; Radscore-2 and DL model-2 were derived from post-nCRT MRI

AUC: area under the curve; DL: deep learning

## Discussion

Radiomics features and DL models based on rectal high-resolution T2WI data pre- and post-nCRT were obtained in this study. Our results demonstrated the merged models combining selected clinical factors, Radscore and DL model predicted the odds of pCR, constituting an effective noninvasive diagnostic strategy for LARC. Furthermore, merged model 1 from pre-nCRT T2WI had AUCs of 0.834 (test set) and 0.746 (validation set), suggesting good predictive efficiency. Clinical decision-making curves demonstrated the preoperative approach conferred added clinical benefit in both test and validation sets.

After nCRT, 15–27% of LARC cases show no live cancer cells, which indicates pCR, and long-term prognosis in such individuals is remarkably improved compared with cases showing residual tumor cells [4]. However, pCR is solely confirmed by pathologic assessment of surgical specimens. Magnetic resonance imaging (MRI)-assessed tumor regression grade (mrTRG) derived from high-resolution T2WI data has been previously introduced prior to surgery, which is a 5-point clinical tumor regression grading system derived from the MERCURY group [4–6]. However, subjective assessment of mrTRG is challenging because of uncertain correlation with pathologic tumor response and the scarcity of consensual treatment response assessment based on MRI [28]. mrTRG 1 shows sensitivity and specificity of only 32.3% and 93.5% for pCR, respectively, while mrTRG 1/2 had values of 69.9% and 62.2%, respectively, as reported in a meta-analysis [28]. Such sensitivity is obviously unsatisfactory.

Multiple reports have demonstrated radiomics represents a promising noninvasive tool with high potential in the prediction of pathological response to nCRT in LARC cases [8–12]. Indeed, a radiomics model showed a higher pooled AUC compared with the clinical radiological model in identifying pCR [9, 16]. Antunes and coworkers [10] developed a pre-nCRT MRI-based radiomics model with an AUC of 0.712 and an accuracy of 70.5%. Peng and collaborators [11] reported AUCs for radiomics models built with pre-treatment, post-treatment, and delta features of 0.771, 0.681, and 0.871, respectively. Wen et al. [12] established a radiomics nomogram with pre-nCRT cN stage, pre-nCRT Radscore and post-nCRT Radscore that achieved an AUC of 0.852, a higher value than obtained with single radiomics models.

Meanwhile, in the field of deep learning (DL), studies have reported DL models show elevated predictive accuracy compared with the radiomics approach, and combined models integrating clinical features have enhanced diagnostic potential compared with radiomics models alone [13–16]. The residual network (ResdualNet) attracts increasing attention from computer vision experts because of high performance. Recently, the residual module is considered the basic module for multiple deep neural networks in the field of biomedical imaging [25–27]. A systematic review [15] reported the algorithm with highest performance had an AUC of 0.845 (range of 0.71–0.99) and an accuracy of 0.85 (range of 0.83–0.98) in imaging prediction of pCR post-nCRT for LARC. Although the DL approach plays an important role in the prediction task, there is still a lack of a more multidimensional and robust preoperative prediction model based on MRI images from pre- or post-nCRT to compare the predictive performance.

It is worth noting that we here compared different radiomics features, DL models and the merged models before or after nCRT. The results indicated the merged model combining baseline clinical factors, pretreatment MRI Radscore and DL model could ameliorate pCR prediction in LARC following nCRT in the test and validation sets. The AUC of merged model 1 was 0.742–0.834, with relatively high sensitivity (0.967–0.978) and accuracy (0.809–0.882). This is consistent with previous findings, and predicting pCR with elevated sensitivity could help noninvasively identify individuals in whom surgical treatment is avoidable, since long-term survival rates are similar in cases with pCR post-rectum resection and the watch & wait group. Consequently, a pretreatment tool was designed effectively to provide an enhanced and objective understanding of predictive models before clinical decision-making.

By comparison, merged model 2 had a relatively low AUC of 0.737–0.746, as well as a relatively low accuracy (0.758–0.815). DCA also revealed enhanced clinical benefits for the pretreatment approach versus the posttreatment model in pCR prediction. These findings indicated that a tumor may exhibit varying degrees of necrosis, edema, scars and fibrosis, which may hamper the accurate segmentation of lesions [29]. Another factor affecting the stability and standardization of results is the variable interval between nCRT and surgery, leading to an inaccurate assessment of tumor heterogeneity, so posttreatment radiomics features and DL model are not sufficient to effectively predict pCR.

Inclusion of an external validation cohort was also notable in this study. When only a few samples are available, especially in the medical field, the model is prone to overfitting. However, because of uniform sample distribution between the training and validation sets, the external validation cohort showed good discrimination, calibration, and performance in the evaluation of pCR. These findings suggest including an external dataset may help mitigate the potential overfitting in a newly developed model. In addition, we adopted data augmentation for guiding and teaching the network to be general and robust. Therefore, the merged model is promising in enhancing diagnostic confidence for radiologists and clinicians in management decisions considering preoperative MRI data.

The current study had limitations. First, manual VOI delineation was employed. Despite its unsuitability for large data, manual segmentation is a more accurate method, which avoids the effect of intestinal wall deformation and potential automatic recognition errors [30–32]. Secondly, although we enrolled an external validation cohort, a retrospective design and a small sample size were used, indicating potential selection bias. Consequently, large multicenter studies are warranted to decrease the effects of data bias on the generalization of the model [33, 34]. Finally, only HR-T2WI for RC analysis was used since other relevant MR sequences, such as CE-MRI, were not available for all patients in our institution. Therefore, we did not apply multiple sequences for analysis to diminish selection bias. Another important aspect was avoiding a large amount of computational burden. Therefore, incorporation of transformer-based model with better suitability for handling large datasets effectively deserves further investigation.

## Conclusions

In conclusion, this study developed pCR prediction models including radiomics and deep learning based on pre- and post- nCRT MRI. Additionally, the pre-nCRT merged model yielded enhanced clinical benefit compared with the post-nCRT model, indicating the model might boost confidence for pCR prediction in clinical management decisions in LARC cases before nCRT.

## Electronic supplementary material

Below is the link to the electronic supplementary material.

Supplementary Material 1

## Data Availability

The datasets used and/or analyzed in the current study are available from the corresponding author on reasonable request.

## Abbreviations

LARC: Locally advanced rectal cancer
TME: Total mesorectal excision
nCRT: Neoadjuvant chemoradiotherapy
TRG: Tumor regression grade
pCR: Pathological complete response
MRI: Magnetic resonance imaging
T2WI: T2 weighted imaging
VOI: Volume of interest
LASSO: Least absolute shrinkage and selection operator
DL: Deep learning
DCA: Decision curve analysis
