# Supplementary material for: Predicting pathological complete response following neoadjuvant chemoradiotherapy (nCRT) in patients with locally advanced rectal cancer using merged model integrating MRI-based radiomics and deep learning data
Source: BMC Med Imaging. 2024 Oct 24;24:289. doi: 10.1186/s12880-024-01474-3 (PMC11515279; doi:10.1186/s12880-024-01474-3)
Supplement: Supplementary file 1 — Supplementary Material 1 [file 12880_2024_1474_MOESM1_ESM.docx]

**Supplementary Materials**

**Supplemental Table S1. T2WI sequence protocol for rectal MRI**

|  | **Training set** | | | **Validation set** |
| --- | --- | --- | --- | --- |
| **Scanner** | **GE**  **Discovery 750w**  **n=92** | **GE**  **Signa HDX**  **n=28** | **SIEMENS**  **MAGNETOM Skyra**  **n=77** | **SIEMENS**  **MAGNETOM Avanto**  **n=52** |
| **Magnetic field strength** | 3.0T | 3.0T | 3.0T | 1.5T |
| **Oblique axial T2WI** |  |  |  |  |
| Echo train length | 32 | 14 | 16 | 15 |
| Field of view (mm) | 200×200 | 180×180 | 180×180 | 180×180 |
| Section thickness (mm) | 4 | 3 | 3 | 3 |
| Matrix | 352×352 | 288×288 | 320×320 | 320×320 |
| TR/TE (ms) | 6538/116 | 4000/113 | 4000/108 | 4000/90 |
| Bandwidth (kHz) | 62.5 | 83.3 | 108/Pixel | 182/Pixel |
| Flip angle (°) | 110 | 90 | 160 | 150 |

TR/TE: Repetition time/echo time.

**Supplemental Table S2. Deep learning algorithm parameter configuration**

| **Pre-processor** | Normalization method | Adaptive Normalizer  min_p: 0.001  max_p: 0.999  clip: True |
| --- | --- | --- |
|  | Equalize sampling | True |
|  | Sampling method | fixed_length |
|  | spacing | [3.0, 3.0, 3.0] |
|  | Crop size | [64, 64, 64] |
|  | Data augmentation | True |
| **Network** | Network model | ResdualNet |
|  |  | Number of training iterations: 1001  Batch size: 8  Number of IO threads: 4  Learning rate: 0.0001  Loss curve update frequency: 100  Epoch Savepoint: 100 |
|  | Learning rate update method | Step  stepSize: 1000  gamma: 0.1  lastEpoch: -1 |
| **Loss** | Loss function | Focal |
|  | Weighting for loss function | 0: 0.5 / 1: 0.5 |
|  | Loss focal gamma | 2 |
| **Optimizer** | Adam | Betas: 0.9/0.999 |

**Supplemental Table S3. Logistic regression analysis of clinical models for predicting pCR**

| **Variables** | **Univariate logistic regression** | | **Multivariate logistic regression** | |
| --- | --- | --- | --- | --- |
|  | OR (95% CI) | P value | OR (95% CI) | P value |
| Gender (male / female) | 1.737 (0.822-3.671) | 0.148 | NA | NA |
| Age (year) | 0.987 (0.954-1.021) | 0.439 | NA | NA |
| Tumor height | 1.000 (0.833-1.201) | 0.997 | NA | NA |
| mr T stage (T1-2/T3-4) | 2.390 (1.089-5.245) | 0.030 | 3.307 (1.233-8.872) | 0.018 |
| mr N stage (N0/N1-2) | 1.912 (1.046-3.494) | 0.035 | 3.883 (1.704-8.847) | 0.001 |
| MRF (negative/positive) | 1.964 (0.832-4.622) | 0.124 | NA | NA |
| EMVI (negative/positive) | 1.694 (0.819-3.604) | 0.155 | NA | NA |
| CEA (negative/positive) | 11.522 (4.520-29.369) | <0.0001 | 14.114 (5.107-39.0074) | <0.0001 |

OR: odds ratio; NA: not available; MRF: mesorectal fascia; EMVI: extramural vascular invasion; CEA: carcinoembryonic antigen (pre-nCRT blood samples).

**Supplemental Table S4. ROC curve analysis of Radscore and DL model in the training set**

|  | **Radscore-1** | **DL model-1** | **Radscore-2** | **DL model-2** |
| --- | --- | --- | --- | --- |
| **AUC** | 0.771 | 0.784 | 0.723 | 0.751 |
| **95% CI** | 0.698-0.834 | 0.711-0.845 | 0.646-0.792 | 0.676-0.816 |
| **Sensitivity** | 0.744 | 0.581 | 0.545 | 0.721 |
| **Specificity** | 0.798 | 0.904 | 0.939 | 0.781 |
| **Accuracy** | 1.000 | 1.000 | 0.994 | 0.873 |

Radscore-1 and DL model-1 were driven from baseline MRI, Radscore-2 and DL model-2 were driven from post-nCRT MRI. Compared by DeLong test, all P values >0.05.

AUC: area under the curve; DL: deep learning.

**Supplementary Figure legends**


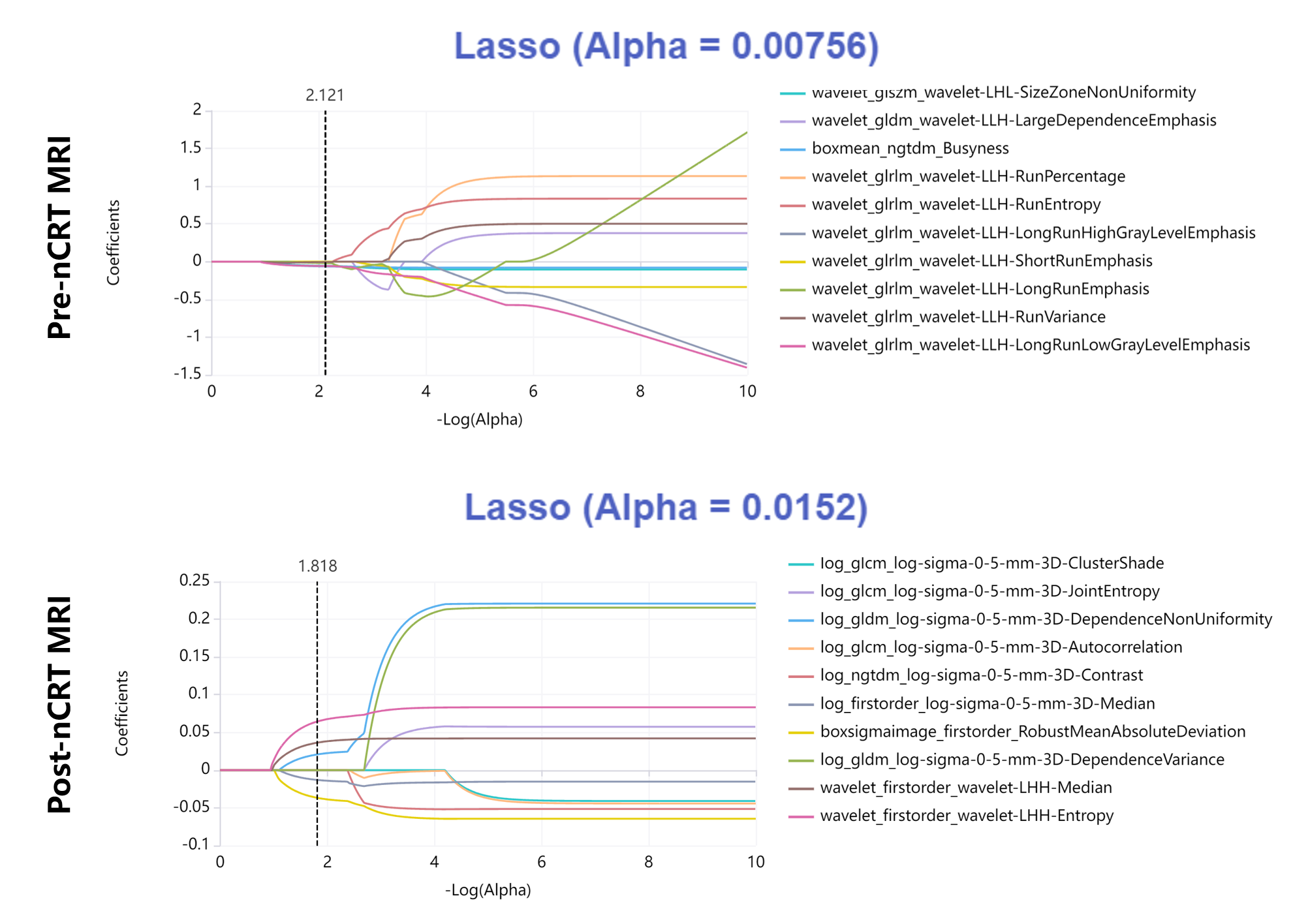


**Supplemental Figure 1.** LASSO paths for feature selection in pre- and post-nCRT MRI.


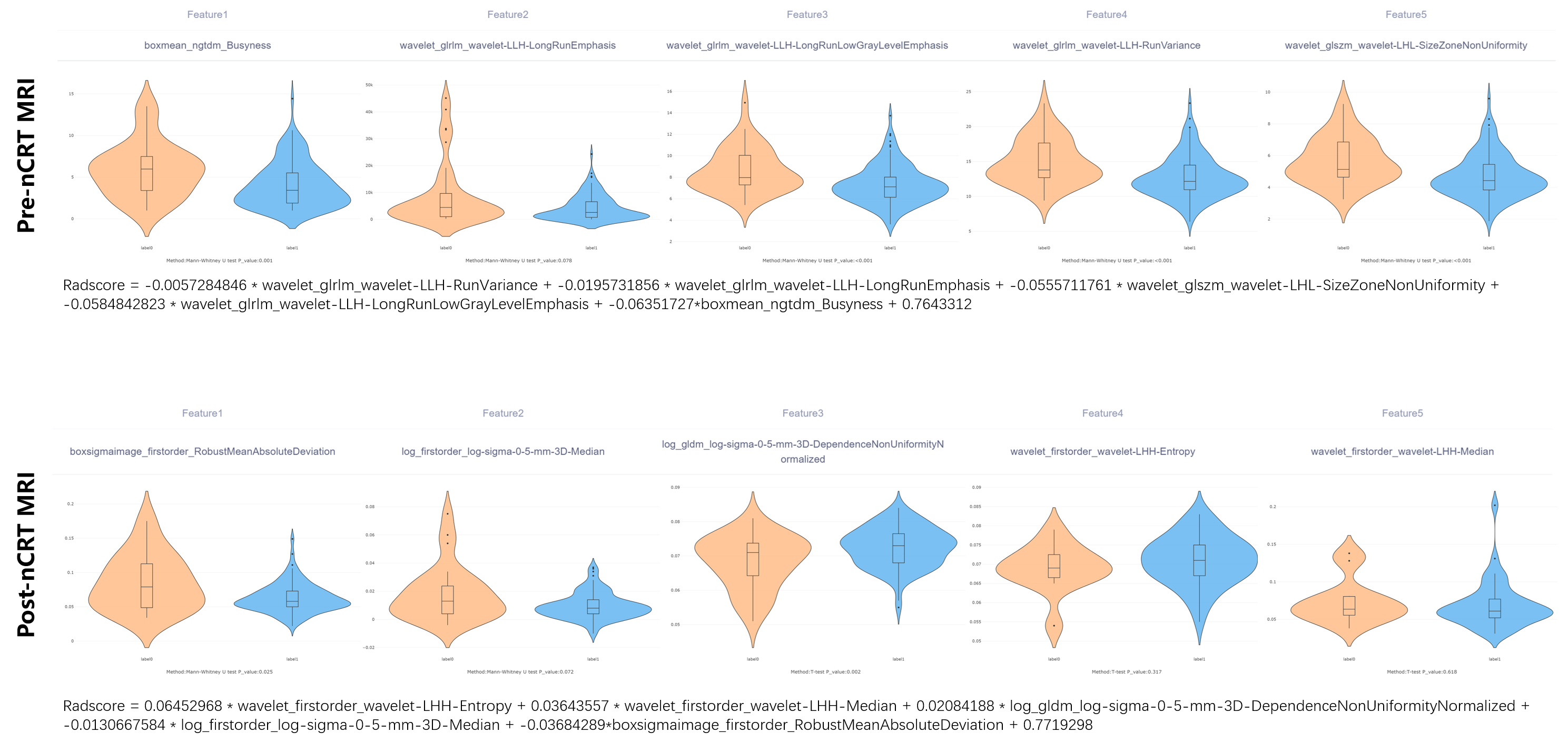


**Supplemental Figure 2.** Violin plots depicting selected features and formulas for Radscore in pre- and post-nCRT MRI.


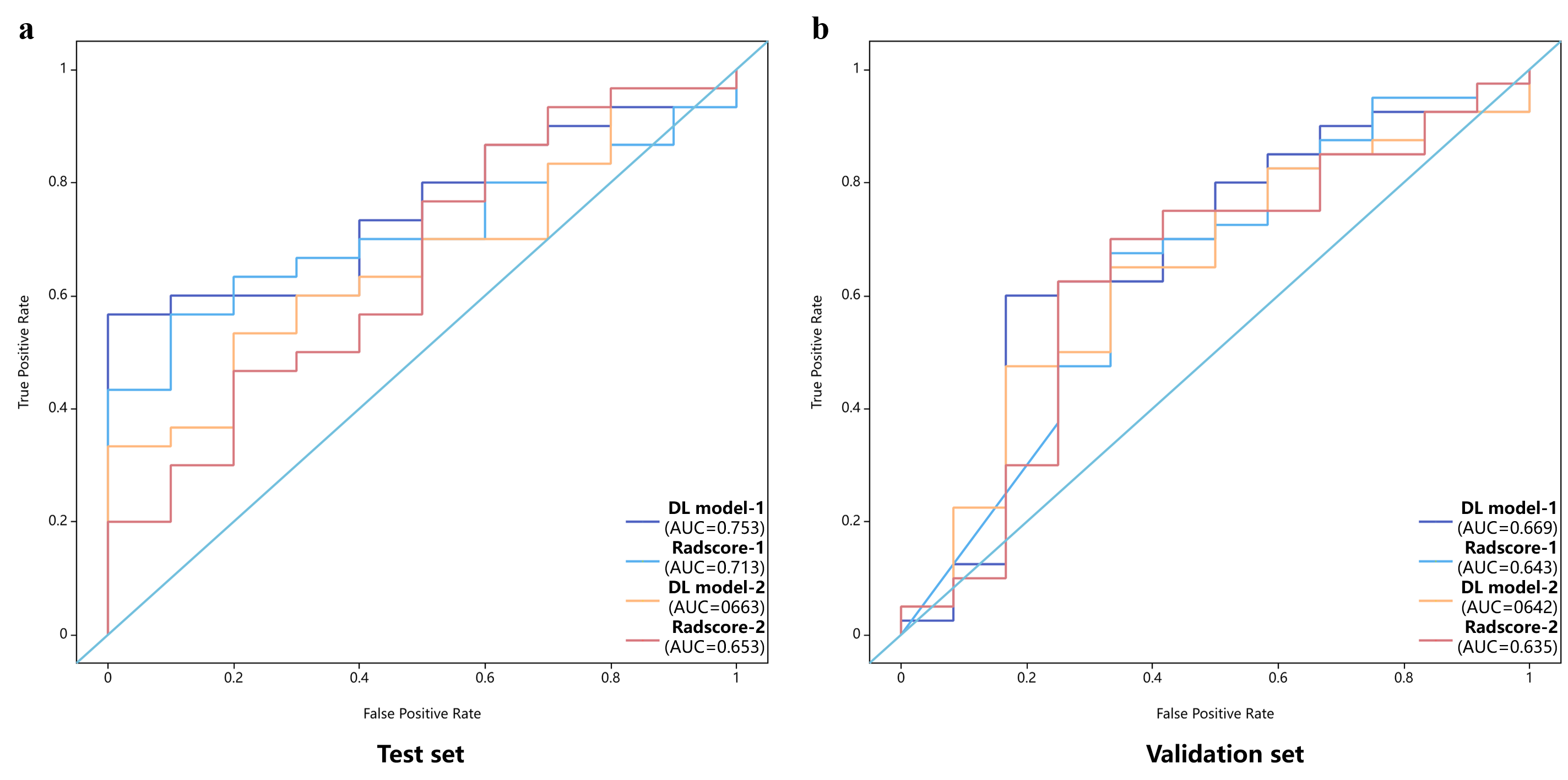


**Supplemental Figure 3.** Receiver operator characteristic (ROC) curve analysis of Radscore and DL model for pCR status prediction. (a) In the test set. (b) In the validation set.


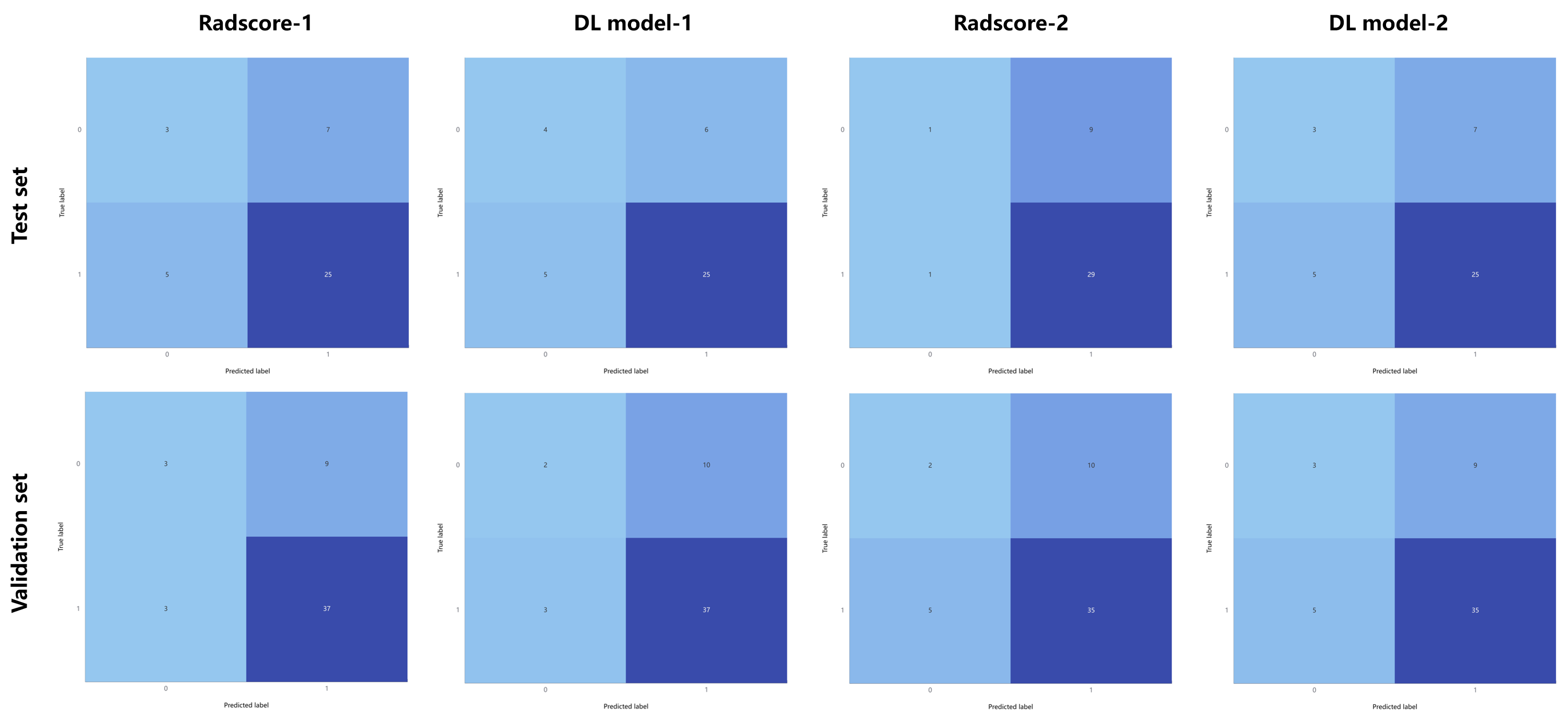


**Supplemental Figure 4.** Confusion matrixes of Radscore and DL models for pCR prediction in the test and validation sets.


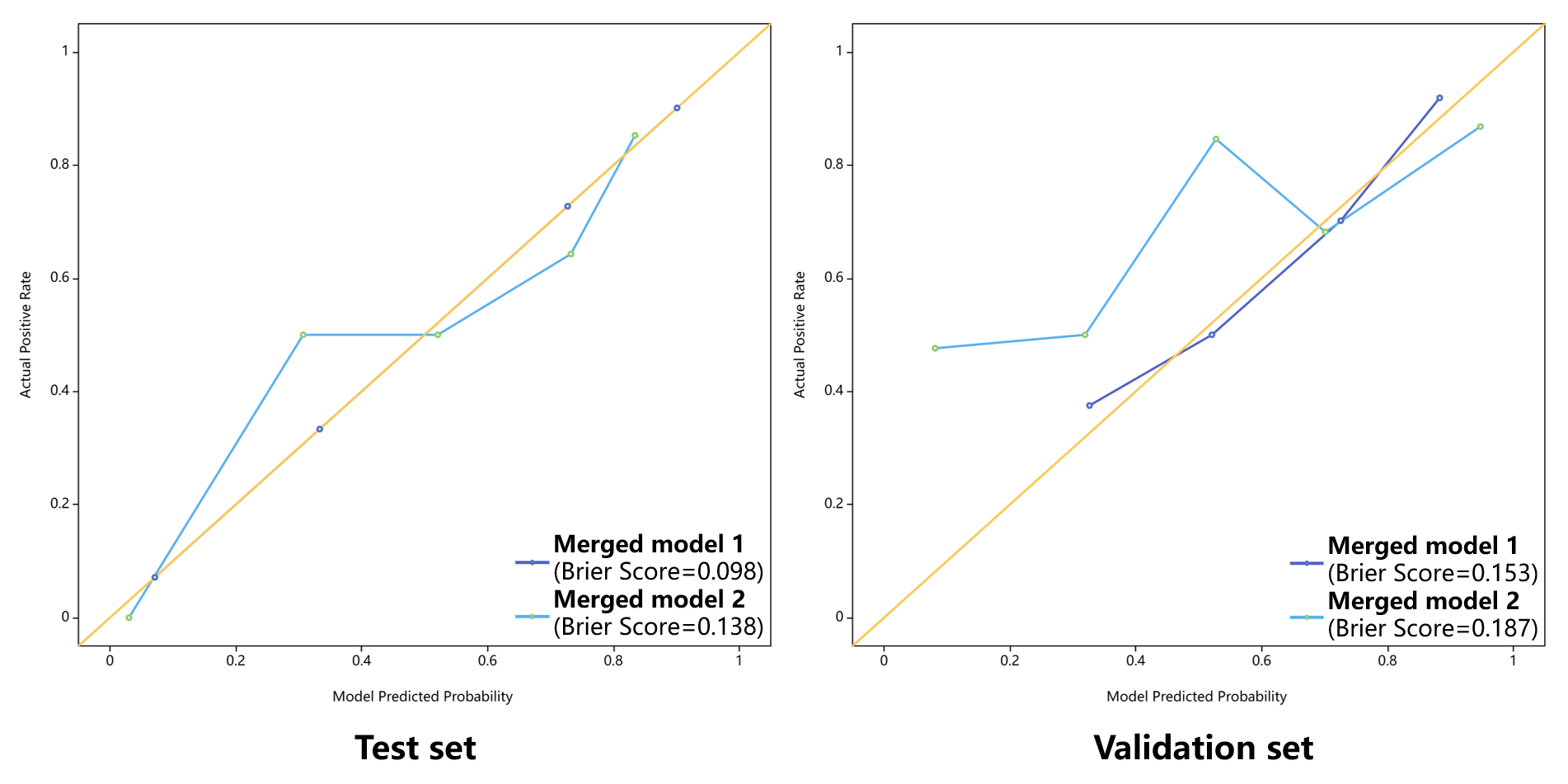


**Supplemental Figure 5.** Calibration curves of merged models 1 and 2 in the test and validation sets.
